# Comparative Investigation of the Influence of Ultrafine-Grained State on Deformation and Temperature Behavior and Microstructure Formed during Quasi-Static Tension of Pure Titanium and Ti-45Nb Alloy by Means of Infrared Thermography

**DOI:** 10.3390/ma15238480

**Published:** 2022-11-28

**Authors:** Elena Legostaeva, Anna Eroshenko, Vladimir Vavilov, Vladimir A. Skripnyak, Arsenii Chulkov, Alexander Kozulin, Vladimir V. Skripnyak, Ivan Glukhov, Yurii Sharkeev

**Affiliations:** 1 Institute of Strength Physics and Materials Science, Siberian Branch Russian Academy of Sciences, 634055 Tomsk, Russia; 2 School of Non-Destructive Testing, Research School of Physics, National Research Tomsk Polytechnic University, 634050 Tomsk, Russia; 3 Department of Mechanics of Deformed Solid Body, Faculty of Physics and Engineering, National Research Tomsk State University, 634050 Tomsk, Russia

**Keywords:** Ti-, Nb-based bioinert alloys, ultrafine-grained state, severe plastic deformation, microstructure, tensile test, mechanical properties, true stress-strain curves, accumulation and dissipation energies, infrared thermography, transmission electron microscopy

## Abstract

A comprehensive study was performed of the deformation and temperature behavior during quasi-static tension, as well as the peculiarities of accumulation and dissipation of energy during plastic deformation. Microstructural analysis at the pre-fracture stage of pure titanium and Ti-45Nb alloy in the coarse grain (CG) and ultrafine-grained (UFG) states was also conducted. It was shown that substructural and dispersion hardening leads to a change in the regularities of dissipation and accumulation energies during deformation of the samples of the pure titanium and Ti-45Nb alloy in the UFG state. Some features of structural transformations during deformation of the pure titanium and Ti-45Nb alloy samples in the CG and UFG states were studied. A band and cellular-network and fragmented dislocation structure was formed in the case of the CG state, while large anisotropic fragments were formed in the UFG state, thus specifying a local softening of the material before fracture.

## 1. Introduction

The development of modern technologies imposes ever-increasing requirements for improving the functional properties of new structural materials. Currently, titanium and titanium-niobium alloys are the key materials and, in many cases, non-alternative materials for strategic industries such as aircraft and rocket engineering, nuclear power engineering, shipbuilding, medicine, food and chemical industries, electronics, etc. [1,2,3].

In medical applications, the materials widely used for manufacturing implants are “pure” α-titanium (commercially pure VT1-0, VT1-00, Grade 1, 2) and (α + β)-medium-strength titanium alloys (Ti–6Al– 4V, Ti–6Al–7Nb, and Ti–6Al–2.5Fe), whose elastic modulus is in the range of 100–120 GPa, which is much higher than the elastic modulus of the cortical bone tissue (10–40 GPa) [2,3,4,5,6,7,8,9,10,11]. According to the literature analysis, a promising direction in medical material science is the development of β-titanium alloys with a low modulus of elasticity close to that of a bone. Doping of titanium with niobium at a concentration of 40–45 wt.% leads to stabilization of the β-phase with a bcc crystal lattice, as well as to a decrease in both the martensitic transformation temperature and the elastic modulus to 50–65 GPa [11,12]. At the same time, the widespread use of “pure” titanium and Ti-(40–45) wt.% Nb alloys in medical practice is limited by insufficiently high strength properties, such as tensile strength and yield strength, hardness, etc. [13,14]. The yield strength of “pure” titanium and the Ti-45 wt.% Nb alloy is 300 and 380 MPa respectively, and their ultimate strength is 400 and 650 MPa [15,16] that is lower than the level of mechanical properties of medium-alloyed titanium alloys for medical applications, such as Ti-6Al-4V, Ti-3Al-5Mo-5V.

One of the ways to solve this problem is the use of severe plastic deformation (SPD) [17]. During SPD, ultrafine-grained (UFG) or nanostructured (NS) states are formed in metallic materials. This leads to a significant increase in structural strength without additional alloying and allows to solve the problem of increasing the mechanical properties [18,19,20,21,22,23,24].

Over the past decades, significant efforts of researchers in many countries have been directed to studying the microstructure and properties of NS and UFG materials [17,18,19,20,21,22,23,24,25,26,27,28,29,30]. A significant difference in the physical and mechanical properties of UFG and NS materials from coarse-grained (CG) materials is associated with the features of their microstructure, namely, with a large volume fraction of non-equilibrium grain boundaries, a high concentration of point and linear defects at near grain boundaries, as well as with a high density of dislocations [31]. The large number of investigations devoted to the study of “pure” titanium (commercially pure VT1-0, VT1-00, commercially pure titanium Grade 1, 2) have shown that the mechanical properties of UFG titanium correspond to medium-alloyed titanium alloys and may replace them. However, despite the large number of publications in this area, intensive studying of UFG metals is still ongoing.

Relatively new for medical applications are Ti-(40–45) wt.% Nb alloys, which have been studied to a fairly lesser extent [32,33,34,35]. This is especially true for the Ti–45 wt.% Nb alloy in the UFG state, since many issues related to the regularities of structure formation for titanium-based alloys with a stabilized low-modulus β-phase under various types of thermal and deformation effects remain scarcely understood and require further systematic analysis.

When developing designs and products for medicine and engineering, it is necessary to analyze the processes of plastic deformation and fracture. It is known that the deformation and fracture of materials are characterized by an increase in the temperature of the plastically deformed material that determines the thermoplastic effect [36]. Energy dissipation during material deformation is accompanied by the transformation of mechanical energy into thermal energy generated by the processes of dislocation motion and annihilation, as well as into the energy of plastic deformation [37,38].

The infrared (IR) thermography [39] has been recognized as an attractive method for studying and analyzing the processes of heat generation under deformation. This technique allows for remotely measuring surface temperature of materials under various conditions and studying the heat generation processes caused by energy dissipation during deformation [40,41,42,43,44,45,46,47,48,49,50,51,52,53,54,55].

At the same time, it should be noted that peculiarities of analyzing deformation heat generation in UFG metals by means of IR thermography have not been well reflected in the literature [45,46,47].

A deeper review of the world publications in the field is beyond the scope of this paper. However, it is worth noting the works [37,38,40,44,45,48,49] which served as a start point for this research. In the above-mentioned publications, it was shown that the method of IR thermography allows efficiently studying the regularities of the evolution of defects in the structure of the material. In [49], the evolution of temperature patterns on the surface of deformable metals during various types of loading was investigated. Respectively, the formation of shear bands at various stages of deformation in the absence of external signs of deformation was revealed to show that energy accumulation and dissipation occur nonlinearly and depend on loading conditions. At the same time, the studies of deformation processes by IR thermography have been carried out mainly on steels, aluminum, magnesium, and titanium alloys in the CG state [50,51,52,53,54,55], and the number of works in which the processes of deformation heat generation in UFG metal materials have been studied by using the IR thermography method is relatively small [45,46,47,56,57].

The peculiarities of temperature distributions during deformation under quasi-static stretching of samples of bioinert alloys VT1–0 titanium, Zr–1Nb and Ti–45Nb in CG and UFG states, including those with structural deviations from “typical samples”, were studied elsewhere in order to identify thermal precursors of deformation and destruction [56]. It was found that the material temperature patterns during deformation and time dependences of the maximum temperature on the sample surface is different for each alloy. They depend on alloy structural state, mechanical characteristics, the presence of structural defects, as well as on alloy thermal properties. It was shown that the presence of macro-defects in samples of VT1-0 titanium, Zr-1Nb, and Ti-45Nb alloys in CG and UFG states, accompanied by reduced strength characteristics, affects the development of deformation and thermal processes in deformable samples. This phenomenon can be effectively visualized by means of IR thermography.

In the recent work [57], the features of deformation under quasi-static stretching of Zr-1Nb alloy samples in CG and UFG states, as well as the distribution of relative deformations and evolution of temperature were studied by digital image correlation and IR thermography methods. The estimation of energy accumulation and dissipation under tension was carried out taking into account the experimental data on thermal processes in the test samples. It has been shown that the differences in the stages of deformation and temperature evolution, as well as the distribution of relative deformations *ε_xx_*, *ε_yy_*, *ε_xy_* in the work zone during stretching of Zr-1Nb alloy samples in the CG and UFG states, are associated with substructural hardening of the matrix phase α-Zr under SPD. 

A drawback of the studies above is the need to study the microstructure, that is formed during deformation loading, and to establish the relationship between the parameters of the deformation structure and the physical and mechanical characteristics of the materials during stretching.

The aim of the present work is to comprehensively analyse the deformation and temperature evolution of alloys under quasi-static tension, as well as the patterns of energy accumulation and dissipation during plastic deformation. The microstructure features after deformation at the pre-fracture stage of VT1-0 titanium and Ti-45Nb alloy in the CG and UFG states have also been investigated.

It should be noted that the novelty of the present work is in the development of a novel approach based on the analysis of deformation, temperature curves and microstructure features in the “neck” formation zone at the stage of pre-fracture. Such approach has allowed to investigate the influence of the UFG structure on the deformation and temperature processes during tension and identify the features of the microstructure after deformation of titanium and Ti-45Nb alloy. This allows a better understanding of the physical mechanisms of deformation and fracture of UFG materials.

## 2. Materials and Research Methods

Commercially pure titanium (99.58 Ti, 0.12 O, 0.18 Fe, 0.07 C, 0.04 N, 0.01 H wt.%) in the UFG state, as well as the Ti-45 wt.% Nb (Ti-45Nb) alloy in CG and UFG states, were investigated. The UFG state in pure titanium and Ti-45Nb alloy was formed by using the combined SPD method, which included the *abc*-pressing with multi-pass rolling followed by pre-recrystallization annealing at 300 °C [25]. To form the CG state, the recrystallization annealing of the UFG samples at 800 °C was used.

The uniaxial tensile mechanical tests of flat samples with a constant strain rate of 0.01 s^−1^ were performed on an Instron VHS40/50-20 test machine (Instron European Headquarters, High Wycombe, UK). The FLIR SC7700M (FLIR Systems, Nashua, NH, USA) thermal imaging system was used to measure sample surface temperature, as well as to evaluate sample size and appearance of a deformation “neck” directly on IR thermograms. This allowed to obtain the temperature Δ*T(ε_true_)* and true deformation *σ_true_*(*ε_true_*) curves and determine the work of plastic deformation *A_p_*(*ε_true_*), the specific amount of the heat dissipated during deformation (*Q)*, and the energy stored during deformation (*Es*). The respective calculations were described in [57].

The total specific work of deformation was estimated using the true strain curves as

(1) $$A=\int_{0}^{\varepsilon_{max}}\sigma_{\mathrm{true}}d\varepsilon_{\mathrm{true}},$$

where *ε_max_* is the maximum deformation of the sample before fracture.

The plastic deformation work (*A_p_)* is defined as

*A_p_ = A − A_e_*, (2)

where *A* is the total mechanical deformation work, and *A_e_* is the elastic deformation work.

The specific quantity of the heat dissipated during deformation (*Q*) was estimated using the parameters estimated thermographically:

(3) $$Q=c\frac{m}{V}\Delta t,$$

where *c* is the heat capacity of the sample material, *m* is the sample mass, *V* is the sample volume, and Δ*T* is the average temperature change on the sample surface under deformation.

The energy stored during deformation (*Es*) is defined as

*E_s_ = A_p_ − Q.* (4)

The microstructure and the phase composition of the samples in the CG and UFG state, as well as under the plastic deformation, were studied using transmission electron microscopy (JEOL JEM 2100, JEOL Ltd., Akishima, Tokyo, Japan). The average size of structural elements (grain, subgrain, fragment) was determined by the secant method [58].

## 3. Results and Discussion

**Figure 1:**
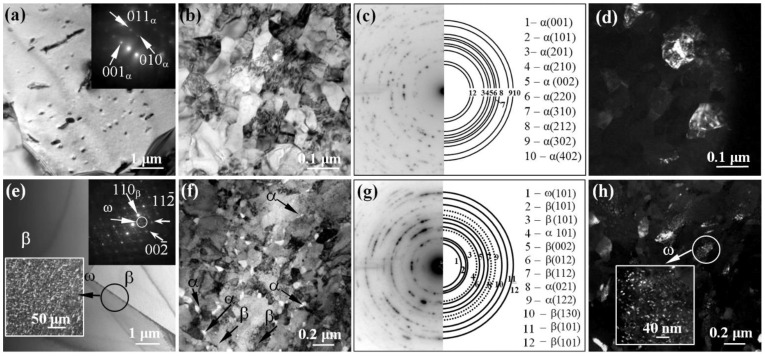
Microstructure of the pure titanium (**a**–**d**) and Ti-45Nb alloy (**e**–**h**) in the CG (**a**,**e**) and UFG states (**b**–**d**,**f**–**h**): (**a**,**b**,**e**,**f**)—the BF TEM images with corresponding SAD patterns; (**d**,**h**)—the DF TEM images. The arrows show the reflections from the identified phases and the phases themselves.

Figure 1 shows the bright-field (BF) transmission electron microscope (TEM) images with corresponding selected area diffraction (SAD) patterns, as well as the dark-field (DF) TEM images of the microstructure of the pure titanium and Ti-45Nb alloys in the CG and UFG states. A typical microstructure of the titanium in the initial state has a coarse-grained structure with equiaxed grains. Randomly located dislocations are observed in the body of the grains (Figure 1a). The identification of the SAD pattern (Figure 1a, inset) revealed the presence of an hcp lattice corresponding to titanium in the α modification. The average grain size was 20 µm.

In the CG Ti-45Nb alloy, the microstructure is represented by matrix subgrains of the β-phase (Figure 1e), and the dispersion-strengthened nanosized ω-phase. Identification of the SAD patterns (Figure 1e, inset) shows the presence of high intensity reflections from the main phase of the solid solution of titanium and niobium (β-phase with a bcc lattice). Formation of the β-phase in the Ti-45Nb alloy is caused by complete stabilization of the bcc crystal lattice due to alloying with the use of a high concentration of an isomorphic stabilizer (niobium) as a result of the α (hcp lattice)→β (bcc lattice) transformation and suppression of the martensite transformation at temperatures below the polymorphic phase transition [59]. A distinctive feature of microdiffraction (Figure 1e, inset) is the appearance of a group of extra reflections of low intensity, which are characteristic of the ω-phase (hp-lattice).

In the DF image obtained in the reflection for the ω-phase (shown by the arrow in the inset, Figure 1e), nanosized particles of the ellipsoidal shape of the ω-phase are observed in the volume of the β-grain. The average grain size of the β-phase was 45 µm, and the segregation of the ω-phase was 0.015 µm (Figure 1e). The presence of the ω-phase is typical for β-titanium alloys. This is due to heat and deformation treatment (conditions at temperatures above the polymorphic transformation), mainly in the regions depleted in the β-stabilizer, niobium. In this case, the β→ω transformation occurs as a result of a non-diffusion mechanism, in which the adjacent atomic planes of the BCC lattice of the β-phase are partially shifted in opposite directions <111> due to the shift [60]. Formation of precisely the ellipsoidal shape of the particles in the ω-phase is associated with a small degree of mismatch between the crystal lattices of the ω- and β-phases [61].

SPD leads to grain refinement and to formation of a grain–subgrain structure in titanium and Ti-45Nb alloy. In the BF and the DF images, there are many extinction contours, which are localized mainly along the boundaries of the subgrains and the fragments (Figure 1b,d,f,h shown by arrows). The observed azimuthal smearing of reflections indicates a high level of internal residual stresses. The SAD patterns (Figure 1g) show the large number of point reflections arranged along circles with typical azimuthal blur, indicating presence of both the high-angle and the low-angle misorientations at the boundaries of subgrains and grains. A microdiffraction analysis of the pure titanium in the UFG state confirmed the presence of the α-Ti phase (hcp lattice). The average size of structural elements of the UFG titanium (grains, subgrains, and fragments) was 0.2 μm. The identification of the SAD pattern for the Ti-45Nb alloy in the UFG state as well as the groups of reflections from three phases were revealed as follows: the high intensity reflections from a solid solution of titanium and niobium (β-phase with a bcc lattice), the low intensity reflections from a non-equilibrium α-phase (hcp-lattice), and the reflections from the ω-phase (ph lattice). As a result of SPD, the α-phase is formed according to the β → α mechanism at the β/β grain boundaries and on the crystal structure defects, the further growth of which occurs on the steps and protrusions of the grain boundaries or according to the ω → α mechanism [62]. According to the ω → α transition, the α-phase appears due to the diffusion of β-stabilizers from the regions with the already isolated ω-phase, or it is formed at the interphase coherent β/ω-boundary [63]. The X-ray spectral microanalysis revealed the presence of subgrains of the β-phase with a high content of niobium, close to the initial state (38–40 wt.%), and subgrains of the α-phase with a low content of niobium (4–10 wt.%). The average size of the structural elements of the β-phase, α-phase, and ω-phase in the UFG Ti-45Nb alloy was 0.2 µm, 0.05 µm, and 0.015 µm, respectively.

The data obtained for alloys in the CG and UFG states by applying the microscopy analysis are presented in Table 1.

**Figure 2:**
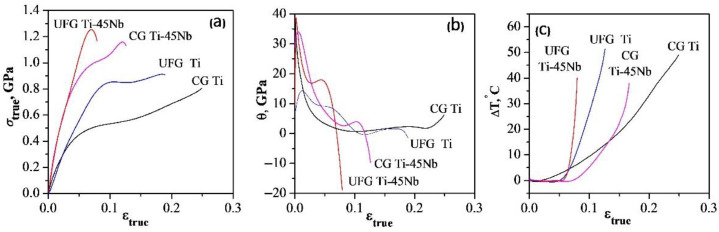
True deformation *σ_true_*(*ε_true_*) curves (**a**), strain hardening coefficient *θ* (*ε_true_*) *= dσ_true_/dε_true_* (**b**) and temperature curves Δ*T*(*ε_true_*) (**c**).

Figure 2 shows the true strain *σ_true_(ε_true_)* curves obtained by considering the neck formation and temperature curves *T(ε_true_)* for pure titanium and Ti-45Nb alloys in the CG and UFG states, and assuming the strain hardening coefficient *θ(ε_true_) = dσ_true_/dε_true_*. For the investigated alloys, there is a significant increase in the true flow stress in the area of neck formation in the curves *σ_true_ (ε_true_)* (Figure 2a). The curve *σ_true_(ε_true_)* for CG titanium has an ascending parabolic section, which turns into a descending section with the *θ* coefficient equal to 5 GPa (Figure 2b, curve 1). In the curves *T(ε_true_)* during the deformation of CG Ti, a short stage is observed with a constant temperature until ε*_true_*~ 0.01 followed by a linear increase of up to about 45 °C before fracture (Figure 2c, curve 1).

The main distinguishing feature of the deformation behavior of UFG titanium is the temperature constancy until ε*_true_*~0.04, indicating its ability to effectively store thermal energy during deformation. With further deformation of UFG Ti, the temperature rises rapidly and reaches Δ*T*~50 °C before fracture in the neck formation zone, which is accompanied by an inflection in the Δ*T(ε_true_)* curve (Figure 2c, curve 2). At the same time, for UFG Ti, the *θ* coefficient before fracture sharply becomes negative, down to (−10) GPa, thus indicating local softening of the material before fracture (Figure 2c, curve 2).

A different picture is observed during plastic deformation of the Ti-45Nb alloy. In this case, regardless of the structural state, a stage with a constant temperature until ε*_true_* ~0.05 is observed, thus proving a greater influence on the deformation and temperature behavior of the dispersion hardening by nanoparticles of the ω-phase, compared to the substructural hardening of the alloy. Above this stage, the temperature grows up sharply, and before destruction Δ*T* reaches ~40 °C for both the CG and UFG states (Figure 2c, curves 1, 2). In addition, the coefficient *θ* before the destruction of the Ti-45Nb alloy becomes negative, and it is equal to (−1.5) and (−20) GPa for the CG and UFG states respectively (Figure 2b, curves 1, 2).

**Figure 3:**
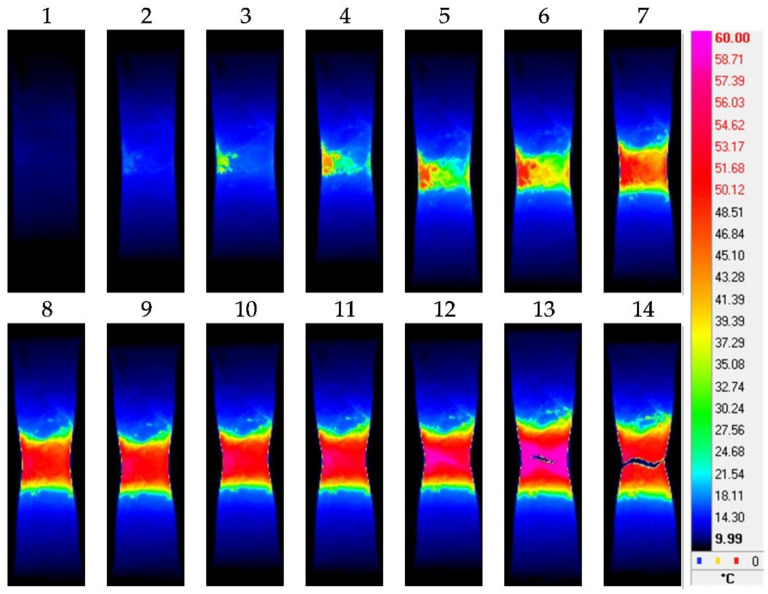
IR thermograms of deformed pure titanium sample in the CG state: 1—*ε* = 0.1; 2—*ε* = 0.11; 3—*ε* = 0.12; 4—*ε* = 0.125; 5—*ε* = 0.14; 6—*ε* = 0.155; 7—*ε* = 0.17; 8—*ε* = 0.18; 9—*ε* = 0.19; 10—*ε* = 0.205; 11—*ε* = 0.215; 12—*ε* = 0.225; 13—*ε* = 0.235; 14—*ε* = 0.24.

**Figure 4:**
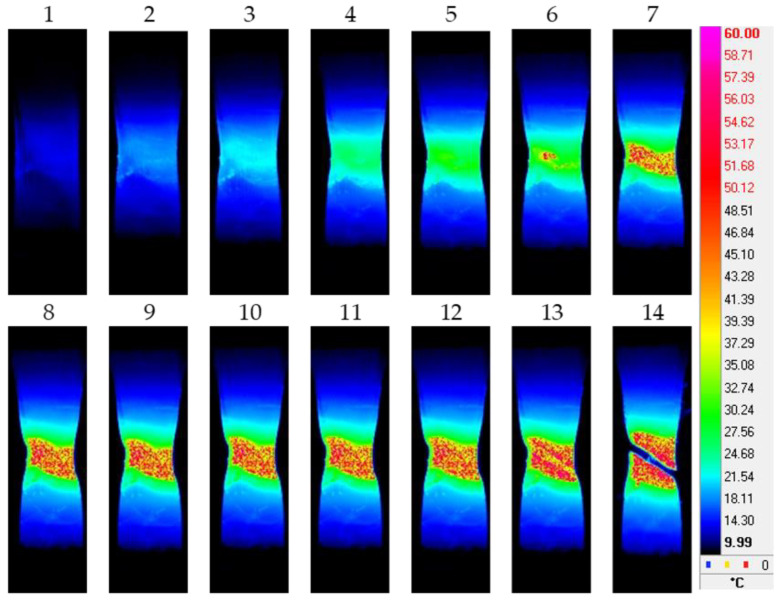
IR thermograms of deformed pure titanium sample in the UFG state: 1—*ε* = 0.05; 2—*ε* = 0.055; 3—*ε* = 0.06; 4—*ε* = 0.065; 5—*ε* = 0.07; 6—*ε* = 0.075; 7—*ε* = 0.08; 8—*ε* = 0.085; 9—*ε* = 0.09; 10—*ε* = 0.095; 11—*ε* = 0.10; 12—*ε* = 0.105; 13—*ε* = 0.11; 14—*ε* = 0.115.

**Figure 5:**
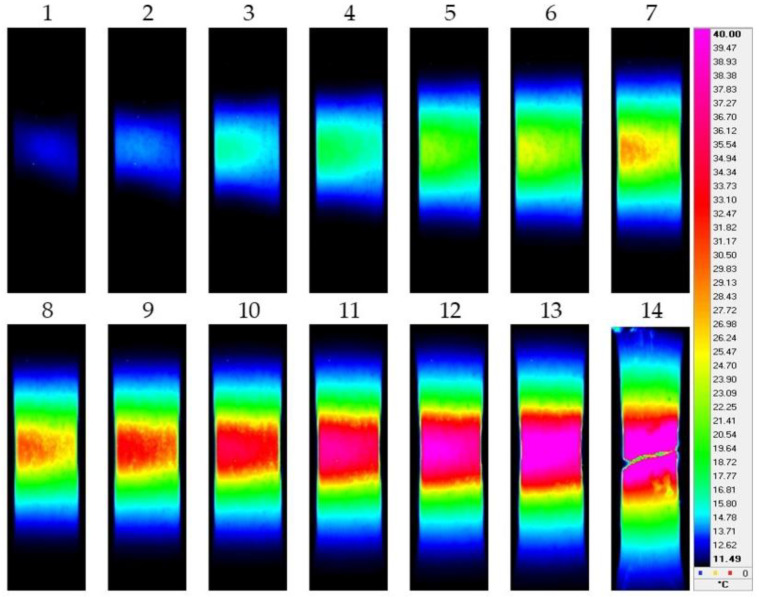
IR thermograms of deformed sample of the Ti-45Nb alloy in the CG state: 1—ε = 0.065; 2—ε = 0.07; 3—ε = 0.075; 4—ε = 0.08; 5—ε = 0.09; 6—ε = 0.1; 7—ε = 0.11; 8—ε = 0.12; 9—ε = 0.13; 10—ε = 0.135; 11—ε = 0.14; 12—ε = 0.15; 13—ε = 0.155; 14—ε = 0.16.

**Figure 6:**
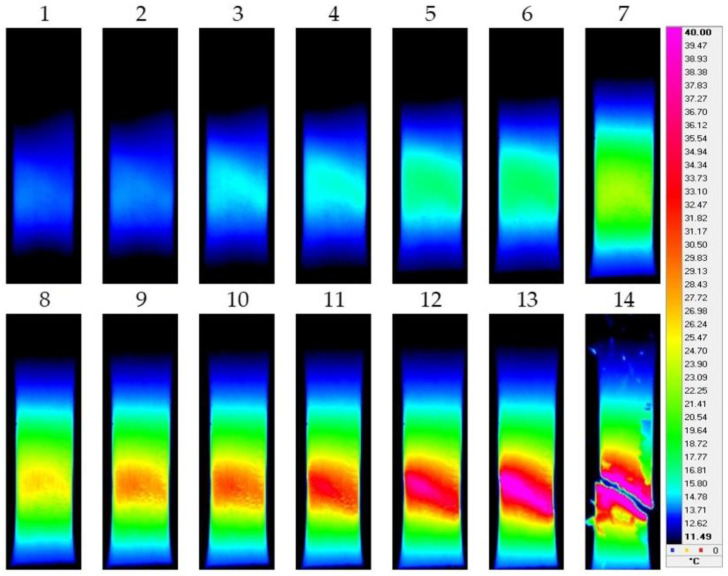
IR thermograms of a deformed sample of the Ti-45Nb alloy in the UFG state: 1—*ε* = 0.055; 2—*ε* = 0.057; 3—*ε* = 0.06; 4—*ε* = 0.062; 5—*ε* = 0.064; 6—*ε* = 0.065; 7—*ε* = 0.066; 8—*ε* = 0.067; 9—*ε* = 0.068; 10—*ε* = 0.069; 11—*ε* = 0.07; 12—*ε* = 0.073; 13—*ε* = 0.075; 14—*ε* = 0.08.

Figure 3, Figure 4, Figure 5 and Figure 6 show the typical IR thermograms images of the thermal distributions obtained by IR thermography during tension of the samples of the pure titanium and Ti-45Nb alloys. It can be seen that during deformation of the pure titanium samples in the CG state in the elastic region, the deformation bands appear in the samples as the sources of heat generation. Their direction corresponds to the highest shear stresses. The generation of the deformation and thermal bands and their development determines location of the defects. The angle of inclination of the bands with respect to the direction of the force during deformation is close to 45°. The deformation and thermal bands divide the sample into blocks. Inside the bands, the metal is in a plastically activated state, whereas in the outer region, the metal operates in the elastic region. As the flow stress increases, the bands width increases. This effect is accompanied by a smooth increase in the sample temperature and the formation of deformation zones.

At the stage of plastic flowing, the deformation centers first increase in size then unite and develop into the form of bands throughout the sample. At maximum exposure in the sample’s weakest place, a pronounced decrease in the cross section occurs to form the neck. Further deformation and the greatest increase in temperature occur in this zone of the sample, and the destruction occurs mainly along the horizontal plane (Figure 3). When the titanium transits into the UFG state, deformation processes become much faster. In the UFG state, the neck in the deformation of the pure titanium is less pronounced, and the destruction of the samples occurs mainly in a plane close to 45° with respect to the direction of the force applied to the sample (Figure 4).

The IR thermograms for the Ti-45Nb alloy in the CG and UFG states indicate that larger deformation centers are generated during the deformation of the samples to serve as sources of heat release. For the Ti-45Nb alloy in the CG state, the width of the deformation bands increases as the flow stress increases, being accompanied by a gradual increase in temperature and the formation of deformation centers, which first increase in size, then unite and develop in the form of a main band. Further deformation as well as the maximum increase in temperature and destruction of the CG alloy occur in the area of the origin of the main band without formation of a neck (Figure 5). At the same time, for the Ti-45Nb alloy in the UFG state, the generation and development of deformation centers proceed much faster, and, before fracture, a sharp jump in temperature is first observed. Afterwards, instantaneous destruction of the sample occurs (Figure 6).

**Figure 7:**
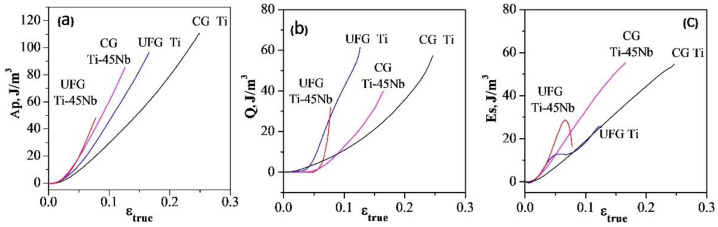
Energy released during deformation as a function of the true strain. *Ap*—plastic deformation energy (**a**), *Q*—heat energy released during deformation (**b**), *Es*—energy stored in the process of deformation (**c**).

Figure 7 demonstrates the relationship between the true deformation and the energy released during deformation of the pure titanium and the Ti-45Nb alloys in the CG and UFG states, namely, the specific work of plastic deformation (*Ap)*, the amount of released heat (*Q)*, and the energy stored during plastic deformation (*Es)*. The limiting specific work of plastic deformation was 110 MJ/m^3^ and 95 MJ/m^3^ for pure titanium and Ti-45Nb alloy in the CG state respectively, and 85 MJ/m^3^ and 48 MJ/m^3^ for these alloys in the UFG state (Table 2). Based on the comparison of the presented dependences, one can conclude that *Ap* for the alloys in the CG state is higher than in the UFG state that is caused by their higher plasticity (Figure 1a).

The analysis of the dependences above shows that the energy *Q* is different for pure titanium and Ti-45Nb alloys in both states. Thus, for CG Ti, the energy released due to the thermoplastic effect is about 50% of the *Ap* value and amounts to 57 MJ/m^3^ (Table 2). The remaining 50% of the energy (53 MJ/m^3^) is stored by the metal.

At the same time, for pure titanium in the UFG state, the *Q* released as a result of deformation is uneven at different stages of deformation. At the initial stage of deformation, until *ε*_true_ ~0.04, almost 100% of *Ap* is stored by the material and turns into the internal energy. Then the dependencies *Q(ε_true_)* and *Es(ε_true_)* are almost linear, and before fracture they experience a sharp jump. In this case, the value of *Q* for the UFG titanium is ~70% of *Ap* (60 MJ/m^3^), and the remaining ~30% of the energy (25 MJ/m^3^) is stored by the metal (Figure 7, Table 2). 

A different picture is observed during plastic deformation of the Ti-45Nb alloy, both in the CG and in the UFG states. At the initial stage of deformation of Ti-45Nb in the CG and UFG states until *ε_true_* ~0.05, almost the whole energy *Ap* is stored by the alloy. This should apparently be assigned to the effect of dispersion hardening by particles of the ω phase with the formation of a new α-phase and substructural hardening of the matrix β-phase. Before the fracture of the CG Ti-45Nb alloy, *Q* increases sharply reaching 40% of *Ap* (40 MJ/m^3^). The remaining 60% of the energy (55 MJ/m^3^) is stored by the material. 

In the UFG Ti-45Nb alloy, *Q* increases abruptly and reaches 32 MJ/m^3^ that is about 70% of *Ap*. Accordingly, *Es* for the UFG Ti-45Nb alloy drops sharply and amounts to ~30% of *Ap* and is 16 MJ/m^3^ respectively (Figure 7, Table 2). This indicates a certain grade of softening of the UFG Ti-45Nb alloy before fracture.

**Figure 8:**
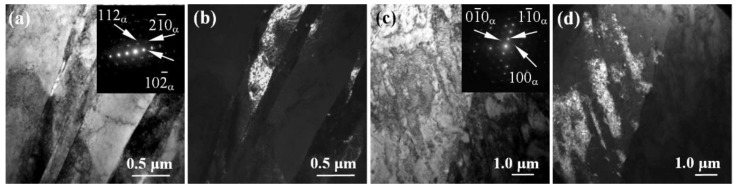
Microstructure of pure titanium in the CG state before fracture in the neck area: (**a**,**c**)—the BF TEM images with SAD patterns; (**b**,**d**)—the DF TEM images. Band structure (**a**,**b**) with cellular-network dislocation substructure (**c**,**d**).

**Figure 9:**
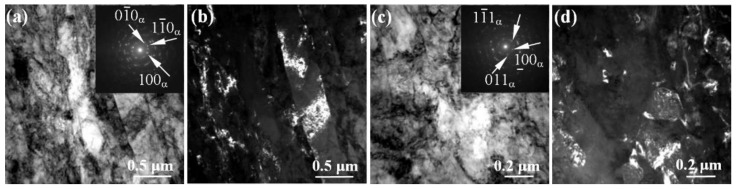
Microstructure of pure titanium in the UFG state before fracture in the neck area: (**a**,**c**)—the BF TEM images with the SAD patterns; (**c**,**d**)—the DF TEM images. Band structure with a cellular-network dislocation substructure (**a**,**b**), large anisotropic fragments with a cellular-network dislocation substructure (**c**,**d**).

**Figure 10:**
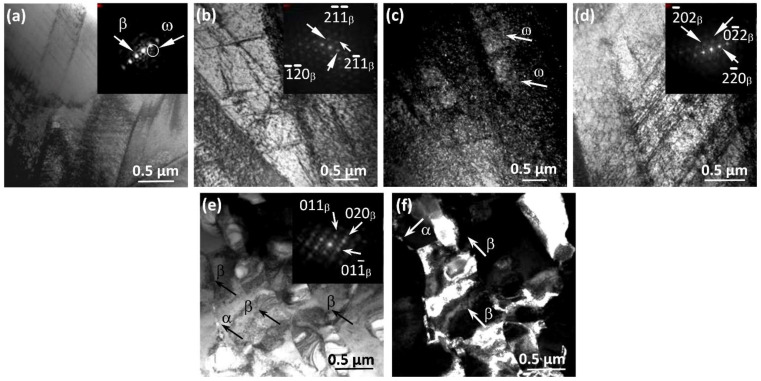
Microstructure of the Ti-45Nb alloy in the CG state before fracture in the neck area: (**a**,**b**,**d**,**e**)—the BF TEM images with the SAD patterns; (**c**,**f**)—the DF TEM images taken in reflections of ω- and β-phases. Band fragments (**a**–**c**) with a cellular-network dislocation substructure (**d**), fragmented substructure (**e**,**f**). Arrows show reflections from identified phases and phases.

**Figure 11:**
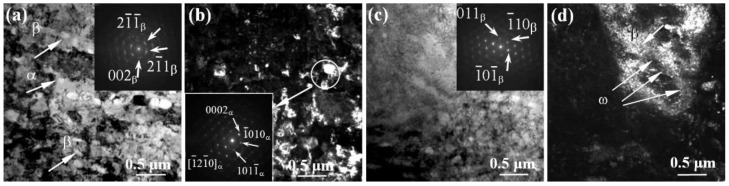
Microstructure of the Ti-45Nb alloy in the UFG state before fracture in the neck area: (**a**,**c**)—the BF TEM images with the SAD patterns; (**b**,**d**)—the DF TEM images taken in (**b**) α-phase reflection and (β + ω)-phase reflection. Fragmented substructure (**a**,**b**), large anisotropic fragments with a cellular-network dislocation substructure (**c**,**d**). Arrows show reflections from identified phases and phases.

Figure 8, Figure 9, Figure 10 and Figure 11 show the microstructure of the studied alloys in the CG and UFG states at the pre-fracture stage in the region of the neck formation. At this stage, the CG microstructure of pure titanium contains a band substructure (Figure 8a,b) consisting of a set of quasi-parallel boundaries of different lengths. The bands have cross-sectional dimensions of 0.2–0.6 μm, whereas their longitudinal dimensions reach 1 μm and more. The TEM images show a high density of bending contours of various shapes, and there are many separate unformed or incompletely collapsed dangling boundaries, which are characterized by multidimensional misorientations (Figure 8c,d). Between the subboundaries in the bands, a high density of dislocations is observed, forming a cellular-network dislocation structure (Figure 8c,d). This substructure can be a result of the accumulation of the dislocation charge in the bands and the occurrence of local long-range stresses, causing a distortion of the crystal lattice due to high dislocation density and strong slip heterogeneity [41].

Before the fracture of UFG titanium, the bands seem to be predominant in the microstructure. The size of the resulting bands in the cross section slightly decreases and drops to 0.3–0.5 µm, whereas their length reaches 1 µm (Figure 9a,b). The bands are formed by collective rearrangement of dislocations with formation of the misorientation boundaries parallel to the subgrain boundary. Triple junctions of grains, that are significant stress concentrators, can also serve as a source of deformation bands. The formation of strain localization bands is the result of relaxation of internal stresses, which arise near the boundaries of fragments and subgrains. Inside, the bands are filled with the regions having a cellular-network dislocation substructure, predominantly with multidimensional misorientations.

It is worth noting that, in comparison with the CG titanium, in the UFG state, along with the band structure, one can also observe anisotropic fragments, i.e., local large volume regions with multidimensional misorientation boundaries (Figure 9c). These areas are represented by a substructure with dislocation walls which are formed due to localization of the dislocations. In the DF images, anisotropic fragments with size of 0.3–0.5 µm are revealed in the indicated areas (Figure 9d). Formation of the anisotropic fragments due to the coalescence of subgrains may indicate a low-temperature deformation return occurring when a critical fragmented structure is reached as a mechanism for relaxation of the accumulated internal stresses [31].

Before fracture, the structure of the Ti-45Nb alloy in the CG state is rather heterogeneous (Figure 10). Two types of structures are formed. First, the areas with band (Figure 10a–d) and fragmented structures (Figure 10e,f) are observed. An increase in the number of locally acting slip systems, as a rule, causes the appearance of a band structure [31,64,65]. The average transverse size of the bands is 0.8 µm, and the longitudinal size is 1.2 µm. A cellular-network dislocation substructure is formed inside the bands (Figure 10b). It should be noted that a cellular-network dislocation substructure is characterized by a fairly uniform distribution of dislocations.

Another type is a fragmented structure, which is formed as a result of further development of the band structure. With increasing magnitude of local internal stresses, the band splits into fragments, and a dislocation cellular-network structure with misoriented volumes is formed inside [31]. As the deformation increases, the density of dislocations in the subboundaries increases. Simultaneously, they become narrower, and fragments with an average size ~0.5 µm are formed. These areas of the substructure are separated by imperfect dislocation boundaries of dislocation clusters accompanied by the presence of a large number of stress concentrators, including precipitates of the ω-phase inside the grain.

In the Ti-45Nb alloy in the UFG state, the microstructure at the pre-fracture stage is of a predominantly fragmented character with low-angle and high-angle boundaries (Figure 11a,b), like in the initial UFG state. Note that the microstructure contains matrix subgrains of the β-phase and grains of the α-phase (along the boundaries and at the joints), as well as segregations of particles of the ω-phase inside the β-grain. The formation of a predominantly fragmented structure may be due to dispersion strengthening by particles of other phases [31]. At the stage of pre-fracture in the UFG Ti-45Nb alloy, as well as in the UFG titanium, the appearance of large (0.9–1.2 μm) anisotropic fragments is observed, inside which a cellular-network dislocation substructure is formed (Figure 11c,d). A specific feature of this structure is a high density of bending contours of various shapes, as well as the patchy contrast and abundance of boundaries (not formed or not completely fractured) with a discrete and continuous set of misorientations [31]. It should be noted that the process of destruction of UFG metals is greatly influenced by their initial defect structure (cells, fragments), grain structure, precipitates at their boundaries and subboundaries, as well as by the grain junctions, internal stresses from boundaries and grain size distribution [31,64,65].

The data obtained as described above was used to compile a classification of the substructures formed during tension of the studied alloys in the CG and UFG states (Table 3).

The main distinguishing feature of structural transformations of the pure titanium samples and Ti-45Nb alloy in the UFG state before fracture, in comparison with the CG state, is the formation of large regions with a cellular-network dislocation substructure and anisotropic fragments. At the same time, during the deformation of the investigated alloys in the CG state, the band and fragmented structures are mainly formed. The formation of large anisotropic fragments also takes place, indicating a local softening of the studied alloys before their fracture.

Thus, the results above indicate that substructural and dispersion strengthening and the formation of the α-phase have a significant influence on the deformation and temperature behavior of the pure titanium and the Ti-45Nb alloy, as well as the patterns of energy accumulation and dissipation during plastic deformations and the type of the emerging substructure during deformation.

## 4. Conclusions

A comprehensive analysis of the microstructure and deformation, as well as the corresponding temperature dependences, demonstrates a significant influence of the UFG state on the regularities of energy accumulation and dissipation during plastic deformation of pure titanium and Ti-45Nb alloy.

It has been found that substructural hardening in UFG pure titanium under SPD leads to changes in deformation, thermal behavior, and energy accumulation and dissipation during deformation. The thermal and energy curves for UFG pure titanium have a plateau (stage up to *ε_true_* ~0.04, at which the total energy of plastic deformation is stored by the metal.

The dispersion strengthening by the *ω*-phase particles and the α-phase formation in the UFG Ti-45Nb alloy reduces the influence of the UFG structure on the deformation and thermal behavior, especially at the initial stage of deformation. At this stage, up to *ε_true_* ~0.05, almost the total energy of plastic deformation is stored by Ti-45Nb in both the CG and the UFG states.

The main distinguishing features of structural transformations during deformation of samples of pure titanium and Ti-45Nb alloy in CG and UFG states were discovered in this study. A band and cellular-mesh and fragmented dislocation structures were formed in the case of the CG state, while large anisotropic fragments were formed in the UFG state. Thus, it indicates a local softening of the material before its fracture.

For a deeper understanding of the deformation mechanisms of pure titanium and Ti-45Nb alloy, it is necessary to investigate the evolution of the microstructure at various stages of plastic deformation. This is the subject of future research.

## Figures and Tables

**Table 1:** Structural parameters and phase composition in pure titanium and Ti-45Nb alloy in the CG and UFG states.

Materials/State	D, μm	Phase Composition, Type Lattice
pure Ti (CG state)	20	α- phase Ti (hcp-lattice)
pure Ti (UFG state)	0.2	α- phase Ti (hcp-lattice)
Ti-45Nb alloy (CG state)	45 0.015	β-phase (Ti,Nb) (bcc-lattice) ω-phase Ti (hp-lattice)
Ti-45Nb alloy (UFG state)	0.2 0.015 0.05	β-phase (Ti,Nb) (bcc-lattice) ω-phase Ti (hp-lattice) α-phase Ti (hcp-lattice)

**Table 2:** Amount of specific energy released during uniaxial tension in the pure titanium and Ti-45Nb alloy in the CG and UFG states.

Materials	*Ap*, MJ/m^3^	*Q*, MJ/m^3^	*Es*, MJ/m^3^
pure Ti (CG state)	110	57	53
pure Ti (UFG state)	85	60	25
Ti-45Nb alloy (CG state)	95	40	55
Ti-45Nb alloy (UFG state)	48	32	16

Here, *Ap* is the energy of plastic deformation, *Q* is the heat energy released during deformation, and *Es* is the energy stored during deformation.

**Table 3:** Types of substructures formed under uniaxial tension in the pure titanium and Ti-45Nb alloy in the CG and UFG states.

Materials/State	Pure Ti CG State	Ti UFG State	Ti-45Nb Alloy CG State	Ti-45Nb Alloy UFG State
Type of substructure	band structure cellular-network dislocation substructure	band structure cellular-network dislocation substructure anisotropic fragments	band structure cellular-network dislocation substructure fragmented structure	fragmented structure cellular-network dislocation substructure anisotropic fragments

## Data Availability

Not applicable.
